# Prevalence of EGFR Mutations in Lung Cancer in Uruguayan Population

**DOI:** 10.1155/2017/6170290

**Published:** 2017-06-28

**Authors:** Nora Berois, Diego Touya, Luis Ubillos, Bernardo Bertoni, Eduardo Osinaga, Mario Varangot

**Affiliations:** ^1^Laboratorio de Glicobiología e Inmunología Tumoral, Institut Pasteur de Montevideo, Montevideo, Uruguay; ^2^Servicio de Oncología Clínica, Hospital de Clínicas, Facultad de Medicina, Universidad de la República, Montevideo, Uruguay; ^3^Departamento de Genética, Laboratorio Epidemiología Genética, Facultad de Medicina, Universidad de la República, Montevideo, Uruguay; ^4^Departamento de Inmunobiología, Facultad de Medicina, Universidad de la República, Montevideo, Uruguay

## Abstract

**Background:**

Incorporation of molecular analysis of the epidermal growth factor receptor (EGFR) gene into routine clinical practice represents a milestone for personalized therapy of the non-small-cell lung cancer (NSCLC). However, the genetic testing of EGFR mutations has not yet become a routine clinical practice in developing countries. In view of different prevalence of such mutations among different ethnicities and geographic regions, as well as the limited existing data from Latin America, our aim was to study the frequency of major types of activating mutations of the EGFR gene in NSCLC patients from Uruguay.

**Methods:**

We examined EGFR mutations in exons 18 through 21 in 289 NSCLC Uruguayan patients by PCR-direct sequencing.

**Results:**

EGFR mutations were detected in 53 of the 289 (18.3%) patients, more frequently in women (23.4%) than in men (14.5%). The distribution by exon was similar to that generally reported in the literature.

**Conclusions:**

This first epidemiological study of EGFR mutations in Uruguay reveals a wide spectrum of mutations and an overall prevalence of 18.3%. The background ethnic structure of the Uruguayan population could play an important role in explaining our findings.

## 1. Introduction

Lung cancer is a leading cause of cancer-related death worldwide, and its incidence makes it a growing public health concern. In Uruguay, it represents the first among males and the third in women, according to the National Cancer Registry report. NSCLC accounts for 80% of primary lung tumors and about one-third of them are diagnosed as locally advanced disease, therefore with poor survival expectancy [1]. Advances in understanding the molecular basis of oncogenesis over the years have led to the discovery of various driver mutations. This has enabled patients' stratification into molecular subgroups exhibiting differential responses to specific therapies. In NSCLC the milestone for such a paradigm shift was the discovery of activating mutations in the EGFR gene, which occurred in 2004 [2, 3]. These genetic alterations, often associated with specific clinical features (histologic type of adenocarcinoma, nontobacco exposure, and Asian ethnicity), are able to identify patients most likely to respond to EGFR tyrosine kinase inhibitors (TKIs) treatments, sometimes with dramatic decrease of tumor growth and significant increase of overall survival. Thus, new standards of care based on personalized therapy applied to clinical management of advanced NSCLC have established TKIs as first-line therapy for patients harboring genetic alterations, leading to improved outcome [4]. However, not all patients benefit equally, and many epidemiologic studies and clinical trials around the world have provided the striking observation that prevalence of EGFR somatic mutations is very different according to ethnic groups and geographic regions, ranging from around 60% in Asian patients to 10–15% in Caucasians [5]. Despite recommendations of major oncology groups to perform EGFR mutation testing to guide therapeutic decisions, this molecular assay is not extensively feasible around the world. A wide variety of technological developments currently in evaluation makes it difficult to establish consensus regarding the optimal detection method for mutational status of EGFR [6]. Although commercially available test kits improved sensitivity and turnaround time, they look only for a preselected set of molecular alterations, so Sanger direct sequencing of DNA is still the “gold standard” enabling identify frequent, uncommon, and novel mutations.

Three compounds which reversibly or irreversibly bind to EGFR (Gefitinib [Iressa, AstraZeneca], erlotinib [Tarceva, Genentech], and afatinib [Giotrif, Boehringer Ingelheim]) have been approved as front-line therapy in EGFR mutant patients, after demonstrating benefit in several large Phase III trials [7]. However, despite an initial dramatic response, virtually all EGFR mutants NSCLC progress as a consequence of acquired resistance. Several approaches to overcome this resistance are under clinical evaluation, including novel and more selective tyrosine kinase inhibitors or combined strategies with inhibitors of multiple pathways. It is important to highlight cumulative evidence suggesting differences in response rates to TKIs depending on EGFR mutation type. At present, guidelines recommend the same treatment for exon 19 deletions and L858R, but several studies have suggested that the former might be more sensitive to EGFR-TKIs than L858R, although the mechanism is still not well understood [8]. On the other hand, other less common mutations, with unknown epidemiology and influence on prognosis and TKI response, have been identified, and more research is required in order to determine their actual significance [9]. Interestingly, EGFR fusions have recently been recognized and also proposed as new therapeutic targets for TKIs [10]. Further investigation, as in vitro diagnostic assays and clinical trials targeting individual EGFR mutations, should give responses in the aim of precision medicine for cancer treatment.

While populations in developed countries have been extensively studied, limited data from Africa and Latin America are available. Uruguay has one of the highest incidence rates of lung cancer (50.1 × 100.000) among Latin American countries [11]. The Uruguayan population, with just only 3.5 million inhabitants, is an admixed population with European, African, and Amerindian genetic contributions [12]. This admixed population structure can affect the mutation distribution of candidate genes with different intensity depending on the disease [13, 14]. EGFR mutation prevalence in Uruguayan NSCLC patients has not been studied earlier, so our aim was to conduct the first epidemiological study in our country.

## 2. Material and Methods

### 2.1. Patients

This study is a prospective analysis of patients who were referred for EFGR testing, from private and public sector hospitals throughout the country, to the Medical Genetics Institute (Italian Hospital, Montevideo) between January 2012 and November 2015 and to the Pasteur Institut of Montevideo from December 2015 to April 2017. The minimal sample size for the study was estimated using the available data in the region [15] and the present in the HapMap. We expected that, with an estimated error of 0.05 and a statistical power of 80%, a sample greater than 250 would be enough to estimate the mutation frequencies in the affected population.

All biologic materials were formalin-fixed paraffin-embedded (FFPE) tissues from primary tumors or metastatic sites, including small biopsies such as transbronchial biopsy and Tru-cut biopsy, surgically resected tumors or cytoblocks from bronchial wash in one case. The inclusion criteria were all cases with a component of adenocarcinomatous differentiation or those in which an adenocarcinomatous component could not be excluded, verified by pathologist before being included in the study. Approval from the Institutional Ethical Committee (Comité de Bioética del Instituto Nacional de Donación y Trasplante de Células, Tejidos y Organos [INDT]) was obtained prior to beginning. All participants provided signed written informed consent previous to enrollment in the study. No other clinicopathologic data were collected for this analysis since samples coming from different institutions throughout the country hinder access to clinical records.

### 2.2. DNA Extraction and Mutational Analysis

All samples were first analyzed in a 3 *μ*m thick hematoxylin-eosin-stained (H&E) slide by a pathologist and tumor-rich areas were marked. In cases showing great stromal cellularity or large necrotic areas, a macro dissection was performed in order to ensure a minimum of 30–40% of tumor cells and over 50% whenever possible. Three 10 *μ*m thick sections were obtained on tubes for DNA extraction using single-use sterilized scalpels, flanked by 3 *μ*m thick cut mounted on slides for H&E staining, in order to estimate actual tumor cellularity. Genomic DNA was extracted by DNeasy Blood & Tissue Kit (Qiagen, Hilden, Germany) according to manufacturer's instructions.

The mutational analysis of exons 18–21 of EGFR gene was performed using nested polymerase chain reaction- (PCR-) based direct sequencing. The first round PCR mixture contains 1x of enzyme provided buffer, 1.5 mmol/L MgCl_2_, 200 *μ*mol/L dNTPs, 400 nmol/L of each primer (primer sequences provided upon request), 1 U* Taq* DNA polymerase (Fermentas, Vilnius, Lithuania), and 50 ng of genomic DNA in a total reaction volume of 20 *μ*L. With the above PCR products as template, a second round of PCR amplification was carried out in the same conditions, but with a total reaction volume of 25 *μ*L, using 400 nmol/L of M13-tailed inner primers. The same PCR cycle conditions for both rounds (carried out in a Biometra Thermocycler) were as follows: initial denaturation step at 95°C for 2 min, 35 cycles of 30 s at 94°C, 45 s at 55°C, and 45 s at 72°C, and a final elongation step at 72°C for 5 min. The final amplicons were visualized on 2% agarose gel after electrophoresis and each band was purified by QIAquick Gel Extraction Kit (Qiagen, Hilden, Germany), then submitted to bidirectional conventional Sanger sequencing (Dye Terminator Cycle Sequencing, ABI PRISM 3700, Applied Biosytems). All sequencing reactions were performed in both forward and reverse directions, and electropherograms were analyzed by visual inspection by an experienced observer and then compared to the available normal sequence in the NCBI database (https://www.ncbi.nlm.nih.gov) using online MultAlin [16]. All variants were confirmed by a second independent analysis of the same DNA source and referred to those published in COSMIC (Catalog of Somatic Mutation in Cancer, http://cancer.sanger.ac.uk/cosmic/).

For statistical comparisons, a Chi-square test was applied. Single-marker allelic and genotypic association tests were performed using the Fisher exact test. A value of *p* ≤ 0.05 was considered statistically significant. The statistical calculations were implemented in R software package version 3.2.3 [17].

## 3. Results

We received a total of 315 tumors for EGFR mutation analysis. Sixteen samples were excluded at first checking due to extensive necrosis or very low tumor cellularity. We successfully amplified all four exons (18 to 21) in 271 cases and three exons in 8 cases. In 13 cases, in which total cellularity was low, final elution volume was reduced, and exons 19 and 21 where prioritized for amplification because of their reported more frequent rate of mutations. In 3 of them, only one exon could be amplified. Taking as inclusion criteria the informative results of at least exons 19 and 21, a total of 289 cases were eligible to conclude about EGFR mutation status. This means a failure rate of 8% (289/315), near the lower boundary of 5–30% reported by the IASLC consensus statement on optimizing management of EGFR mutation positive NSCLC, recently updated [5].

**Figure 1:**
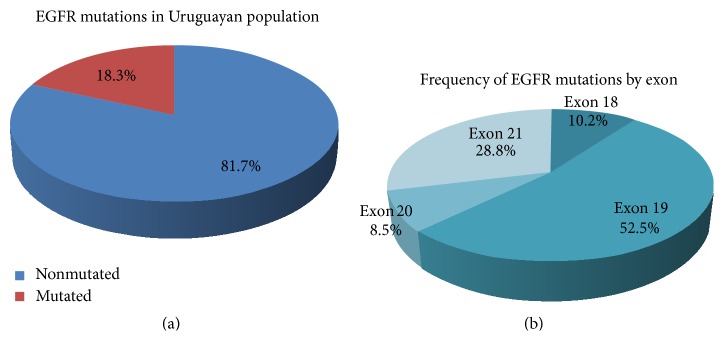
(a) shows overall EGFR mutations in Uruguayan population. (b) shows distribution by exon of EGFR mutations.

**Figure 2:**
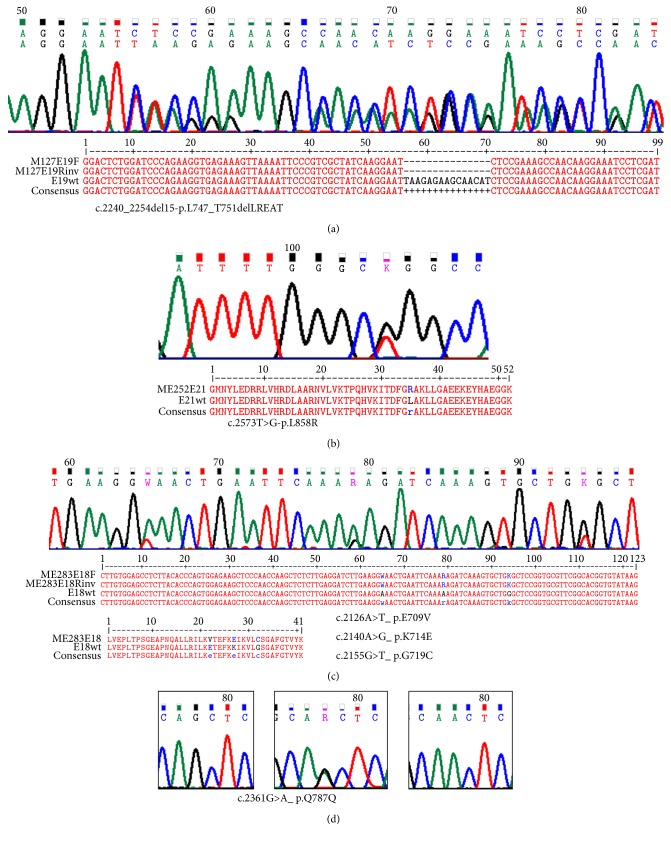
Examples of observed mutations: (a) exon 19 deletion; (b) missense mutation L858R in exon 21; (c) simultaneous triple point mutations in exon 18; (d) the most frequent coding silent substitutions Q787Q.

Out of 289 studied cases, 165 were males and 124 were women. Overall EGFR mutations were detected in 53 of the 289 patients (18.3%) (Figure 1(a)), more frequently observed in women 29/124 (23.4%) than in men 24/165 (14.5%) (*p* = 0.127, Chi-square test of independence). Four patients had multiple mutations, so a total of 59 mutations were distributed by exon as shown in Figure 1(b): 6/59 (10.2%) in exon 18, 31/59 (52.5%) in exon 19, 5/59 (8.5%) in exon 20, and 17/59 (28.8%) in exon 21.  Table 1 shows all found mutations with modified translation. The main types were simple or complex in-frame deletions in exon 19 (30/59, 50.8%) (Figure 2(a)), followed by the missense substitution L858R in exon 21 (13/59, 22.0%) (Figure 2(b)). Several uncommon mutations were found through the 4 exons, most of them point mutations (Table 1). Four patients showed multiple mutations: one case showing a point mutation in different exons (p.G719C in exon 18 plus p.S768I in exon 20), one case showing a simultaneous triple point mutation in the same exon 18 (p.G719C, p.K714E and p.E709V) (Figure 2(c)), another case with a triple mutation in exon 18 (p.E711G), exon 19 (p.E746_A750del), and exon 20 (p.V769M), and one case with the double mutation p.E746_A750del in exon 19 plus T790M in exon 20. This patient was previously treated with TKIs and the resistance mutation T790M was certainly selected under treatment pressure. Another patient showed de novo T790M. The most frequent uncommon mutations were p.P848L and p.G719C; each one was found twice. We also found twice a point mutation at p.K714, although the amino acid substitution was different. While p.K714N was reported, the other one (p.K714E) was not reported in COSMIC.

Various coding silent substitutions (nucleotide change without amino acid change in the EGFR protein) were observed, mainly in exons 20 and 21 (Table 2). The most frequent was c.2361G>A-p.Q787Q (Figure 2(d)), followed by the less common c.2508C>T-p.R836R, both reported in COSMIC. To ascertain a relationship between c.2361G>A-p.Q787Q polymorphism and the missense mutations or exon 19 deletions, we performed an association analysis and no significant values were found in any of the mutations (Table 2). Interestingly, three other silent substitutions, not previously described, were also observed: c.2331G>A- p.L777L; c.2547G>A-p.Q849Q; and c.2565 T>C-p.D855D (Table 2).

## 4. Discussion

**Figure 3:**
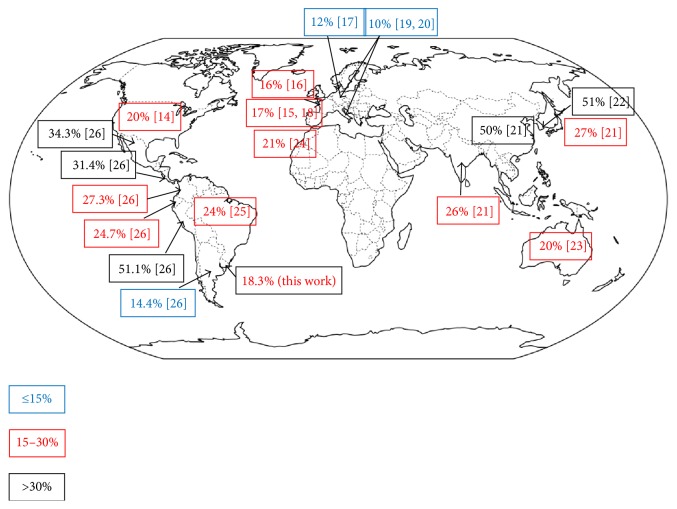
Worldwide distribution of EGFR mutations.

Differences in EGFR somatic mutation frequency among ethnic groups and geographic regions have been largely reported [18–31] (Figure 3). The Asian population shows the higher prevalence (50–60%), but in Caucasians only 10–17% is observed. Those populations have been extensively evaluated, while in Latin America limited data have been published, and for African populations results are controversial. To our knowledge, excepting a single study from Morocco, showing a frequency of mutations of 21%, similar to Caucasian people [29], most data concerning African populations come from limited-sized cohorts of self-defined racial groups, conducted mainly in the United States. While first reports account for an EGFR mutation frequency as low as 2% [32], other reports show discrepant data of 12% and 19% [33, 34]. Clifford et al. reviewed those controversial reports, pointing at some potential reasons: population bias concerning sex, smoker status and self-reporting ethnicity, disparity in technological approaches used to detect mutations as well as Simpson's paradox, which is the statistical correlation observed in aggregated heterogeneous groups which could be reversed when groups are disaggregated [35]. Moreover, in a recent study in Caribbean populations, mostly African descendent, Leduc et al. found EGFR mutations in 36% of patients, which is higher than expected [36]. The authors ascribed their finding to environmental factors such as low tobacco consumption. More research is absolutely required in order to clarify EGFR mutation prevalence in the African population.

On the other hand, Arrieta et al. published in 2011 a brief report on genotyping NSCLC in Latin America, showing EGFR mutations' prevalence for Argentina, Colombia, Mexico, and Peru [15]. This study was updated in 2015, expanding the number of cases and including data from Panama and Costa Rica [31]. This is the largest study reporting data for Latin American patients (*n* = 5738), although it should be pointed out when comparing data that only exons 19 and 21 were analyzed by direct sequencing for Argentina, Colombia, Peru, and Costa Rica, while in Mexico and Panama all four exons were evaluated for known mutations by a sensitive commercial kit based on ARMS technology. The study highlights a wide range in EGFR mutation frequency, ranging from 14.4% for Argentina, 24.7% for Colombia, 27.3% for Panama, 31.4% for Costa Rica, 34.3% for Mexico up to 51.1% for Peru. This supports the genetic heterogeneity of NSCLC among geographic regions and also underscores the need for extending the study to other countries of South and Central America. Several studies conducted in Brazil reported 24% of EGFR mutation in adenocarcinomas [30], in agreement with De Melo et al., who reported 21.6% [37]. Interestingly, in this study most are rare mutations, while common mutations in exon 19 and 21 represent only 40% of the total mutations. In contrast, Carneiro et al. reported only 6.6% of EGFR mutations in lung adenocarcinoma [38]. These authors evaluated ancestry among tumors and healthy controls and found an African component more prevalent in lung cancer than in controls.

Our results show a prevalence of EGFR mutations in the Uruguayan population of 18.3%, which is somewhat higher than in Europeans but lower than in other Latin American countries, which have a mutation prevalence value intermediate between Asian and Europeans. A possible explanation could be a different genomic ancestry among Latin American populations. Native Americans arise from a single (or even two) migration wave from Asia 20.000 years ago [39, 40]. However, after the European and African arrivals, regional scenarios in ethnic, cultural, and social relationships led to a complex process of admixture which makes unique the genetic composition of Latin American population [41]. This ancestral genome may be an explanation for the differences found in reported EGFR mutation frequency in different Latin American countries. For example, the higher prevalence found in Peru may be related to the prevalent Amerindian ancestry (ranging 76–98%), with 1–31% of European ancestry and 1–3% of African ancestry [42]. In Mexico, Amerindian ancestry accounts for 51–56%, European 40–45%, and African 2–5%, which may be in concordance with the reported 34.3% of EGFR mutations. Uruguayan population has been classically described as essentially of European origin, mostly from Spain and Italy. However, genetic admixture analysis has shown that it is a trihybrid population, with genetic contributions from Native Americans (10.4%) and Africans (5.6%) [43], which could explain our slightly higher than expected results.

Most authors agree that the two most common EGFR mutations (exon 19 deletions and L858R), accounting for 80–90% of all mutations, are the best predictors for TKIs response, although exon 19 deletions showed significantly better outcome than L858R point mutation [44]. However, much less is known about the significance of uncommon mutations. In our series several rare mutations have been identified (Table 1). Among them, exon 18 mutations p.G719C and p.E709X have been reported as sensitive to afatinib and neratinib [45]. We also found 2 mutations at p.K714. One of them (p.K714N) was described by Locatelli-Sanchez et al. [21], but the other one (p.K714E) was not previously reported. Interestingly, these authors reported near 10% of previously not described mutations as well as several concurrent mutations in French population. Some of these mutations were at the same locations as our series, for example, a concurrent triple point mutations in exon 18 (p.E709, p.K714, and p.G719) and a concurrent deletion in exon 19 plus the point mutation p.V769M in exon 20. We also found a complex deletion in exon 19 (p.E746_T751del>FPS) as well as a codon insertion in exon 20 (c.2308_2309insTGG; p.V769_D770insV), both of them not described in COSMIC. The resistance mutation T790M in exon 20 was found in two cases, one de novo mutation and the other one being probably selected under treatment pressure, since this patient was previously treated with TKIs. Other uncommon described mutations found in our series (p.L861Q and p.768I) have been suggested as less sensitive for first-generation TKIs [46], but in vitro experimental research suggests sensitivity for second- and third-generation TKIs [47]. Lastly, some very rare mutations in exon 21 were observed: p.P848L (*n* = 2), reported as a resistant to TKIs germ-line mutation by Prim et al. [48], and p.A840T (*n* = 1), also showing no benefit from TKIs [49].

Concerning coding silent substitutions (Table 2), we observed a high frequency of c.2361G>A_p.Q787Q in exon 20. This exon could be successfully sequenced in 273 cases and the G allele has a frequency of 41%, which is in agreement with those published for Latin American populations (45%) in the 1000 Genomes Project database [50]. These admixed populations (Colombia, Peru, Mexico, and Puerto Rico) clearly show an allele frequency that ranges between the Asian (82%) and European (39%) sample population frequencies. Very few papers reported this polymorphism in EGFR mutations studies. Carneiro et al. found a slight difference with respect to healthy controls in a Brazilian study for this SNP; however, this finding needs to be taken with caution due to the sample size and the admixture structure of the samples [38]. Zhang et al. described it as more frequent in tumors compared with healthy controls [51], but independently of other EGFR mutations. Little is known about its eventual clinical significance. Sasaki et al. found this polymorphism less frequent in the Japanese population, which is in concordance with the dbSNP database, and more frequent in other histological types than in adenocarcinoma [52]. These authors also suggest a tendency toward better outcome in wild-type patients. Recently Koh et al., studying another Asiatic population, suggested, in agreement with Sasaki et al., that it could be an independent prognostic marker for low stage lung cancer patients [53]. In our study, we found no association of this marker with the most important mutations, p.L858R, and exon 19 deletions. The polymorphism c.2361G>A-p.Q787Q seems to be related to the geographic susceptibility of mutations in EGFR patients, higher in Asians than Europeans [5].

We also found another known polymorphism, c.2508C>T-p.R836R, in 8/289 cases (2.8%). According to the 1000 Genome database, this polymorphism is found from 0 to 2% in European, Asian, Latin American, or even African populations, but it has been reported in colorectal cancer, breast cancer, and head and neck cancer. In lung cancer, such polymorphism was reported by Schmid et al. in a study looking for correspondence in mutational status between primary tumors and lymph node metastasis in a subset of Austrian patients [54]. They found the Q878Q polymorphism in 83% of both primary tumors and metastasis, but the R836R polymorphism was found only in 4/96 (4%), mainly in metastasis, with only one case showing concordance with the primary tumor. Moreover, we observed three other still nondescribed coding silent substitutions at codons 777, 849, and 855 (Table 2).

In conclusion, this first epidemiological study of EGFR mutations in the Uruguayan population reveals a wide spectrum of mutations and an overall prevalence of 18.3%, which indicates that the background ethnic structure of the Uruguayan population plays an important role in explaining the observed data. More profound studies are needed to understand the implication of ethnic background in lung cancer development of the Uruguayan population.

## Figures and Tables

**Table 1:** Mutations in the EGFR gene.

*n*	Sex	Mutations				
		Type of mutation	Exon	Mutation ID (COSMIC)	Nucleotide change	Amino acid change
1^*∗*^	F	Substitution, missense	18	—	c.2132A>G	p.E711G
2^*∗*&^	M	Substitution, missense	18	COSM6253	c.2155G>T	p.G719C
1^*∗*^	M	Substitution, missense	18	COSM12371	c.2126A>T	p.E709V
1^*∗*^	M	Substitution, missense	18	—	c.2140A>G	p.K714E
1	M	Substitution, missense	18	COSM1651578	c.2142G>T	p.K714N
1	M	Substitution, missense	19	COSM52931	c.2195T>C	p.I732T
6	F	Deletion, in-frame	19	COSM6223	c.2235_2249del15	p.E746_A750delELREA
3	M	Deletion, in-frame	19	COSM6223	c.2235_2249del15	p.E746_A750delELREA
3^&^	M	Deletion, in-frame	19	COSM6225	c.2236_2250del15	p.E746A_750delELREA
7^*∗*^	F	Deletion, in-frame	19	COSM6225	c.2236_2250del15	p.E746A_750delELREA
1	F	Deletion, in-frame	19	COSM6254	c.2239_2253del15	p.L747-T751delLREAT
1	M	Deletion, in-frame	19	COSM12369	c.2240_2254del15	p.L747_T751delLREAT
1	M	Deletion, in-frame	19	COSM13556	c.2253_2276del24	p.S752_I759delSPKANKEI
5	F	Complex, deletion in-frame	19	COSM12370	c.2240_2257del18	p.L747_P753del>S
2	M	Complex, deletion in-frame	19	COSM12382	c.2239_2248del10>C	p.L747_A750del>P
1	F	Complex, deletion in-frame	19	—	c.2235_2251del17>ATTCCCGT	p.E746_T751del>FPS
1^&^	M	Substitution, missense	20	COSM6241	c.2303G>T	p.S768I
1^*∗*^	F	Substitution, missense	20	COSM13425	c.2305G>A	p.V769M
1	F	Insertion, in-frame	20	—	c.2308_2309insTGG	p.V769_D770insV
2^&^	M	Substitution, missense	20	COSM6240	c.2369C>T	p.T790M
2	F	Substitution, missense	21	COSM22943	c.2543C>T	p.P848L
1	M	Substitution, missense	21	COSM85961	c.2518G>A	p.A840T
6	F	Substitution, missense	21	COSM6224	c.2573T>G	p.L858R
7	M	Substitution, missense	21	COSM6224	c.2573T>G	p.L858R
1	M	Substitution, missense	21	COSM6213	c.2582T>A	p.L861Q

^*∗*^Triple mutation in the same patient. ^&^Double mutation in the same patient.

**Table 2:** Coding silent substitutions in the EGFR gene.

*n*	Substitution, coding silent					
		COSMIC ID	Nucleotide	Amino acid	rs	Location
1	AA	—	c.2331G>A	p.L777L	—	Chr7: 55.181.340
217	AA = 108	COSM1451600	c.2361G>A	p.Q787Q	rs1050171^*∗*^	Chr7: 55.181.370
	GA = 109					
8	TT = 1	COSM85893	c.2508C>T	p.R836R	rs2229066	Chr7: 55.191.757
	CT = 7					
1	AA	—	c.2547G>A	p.Q849Q	—	Chr7: 55.191.796
1	TC	—	c.2565 T>C	p.D855D	—	Chr7: 55.191.814

^*∗*^Allele G = 0.405. Hardy-Weinberg Equilibrium *p* value = 0.139. Association test, p.L858R (*p* value = 0.239) and exon 19 deletions (*p* value = 0.296).

## Abbreviations

EGFR: Epidermal growth factor receptor
NSCLC: Non-small-cell lung cancer
TKI: Tyrosine kinase inhibitors
FFPE: Formalin-fixed paraffin-embedded
COSMIC: Catalog of Somatic Mutation in Cancer
NCBI: National Center for Biotechnology Information.
